# Case report: Submucosal cavernous lymphangioma causing jejuno-jejunal intussusception in an adult

**DOI:** 10.3389/fsurg.2022.953840

**Published:** 2022-09-22

**Authors:** Ning Zhao, Yuhang Fu, Zhongzheng Wang, Qi An, Wenzhuo Jia

**Affiliations:** ^1^ Department of General Surgery, Department of Gastrointestinal Surgery, Beijing Hospital, National Center of Gerontology, Institute of Geriatric Medicine, Chinese Academy of Medical Sciences, Peking Union Medical College, Beijing, China; ^2^ Department of General Surgery, Peking University Fifth School of Clinical Medicine, Beijing Hospital, Beijing, China

**Keywords:** cavernous lymphangioma, intussusception, jejunal tumor, case report, small intestine

## Abstract

Cavernous lymphangioma often occurs in the head, neck, trunk, and extremities of infants and children, and it is rare to cause a small intestine intussusception in adults. In this case, a 32-year-old woman presented with abdominal pain, vomiting, and a 5 cm × 5 cm abdominal mass on the left side of the abdomen. Laboratory tests showed anemia and CT showed small intestinal intussusception. After conservative treatments, her symptoms disappeared. However, 18F-FDG PET/CT suggested malignancy and her symptoms reappeared after eating something. Segmental jejunal resection was performed and pathology showed submucosal cavernous lymphangioma. At the 1-year follow-up, the patient was asymptomatic. Then this paper reviewed the literature on small intestinal cavernous lymphangioma in adults and found that this is the first English case report of intussusception caused by a jejunal submucosal cavernous lymphangioma in an adult. Current problem is that adult intussusception and intestinal lymphangioma are difficult to diagnose preoperatively. Imaging techniques such as tomography and PET/CT aid in the diagnosis of these benign lesions. Surgical resection was considered to be the required treatment and seems to have had no recurrence in adults according to the literature.

## Introduction

Intussusception caused by small intestinal cavernous lymphangioma in adults is rare. The etiology of intussusception in adults is often benign polyps, Meckel's diverticulum, malignant lesions, or idiopathic lesions, which account for approximately 1%–5% of mechanical intestinal obstruction and are malignant in up to 77% of cases (1, 2). Lymphangioma is a malformation of the lymphatic vessels and is a benign tumor that often occurs in the head, neck, extremities, and trunk region with an incidence of 1: 2,000–4,000, and most patients (80%–90%) are diagnosed before the age of two years (3). Lymphangiomas account for 6% of small intestinal tumors in children (4) and approximately 1.4%–2.4% in adults (5). Abdominal lymphangiomas account for about 5% of lymphangiomas, often in the mesentery and about 5% in the retroperitoneum (6, 7). The incidence of cavernous lymphangioma is not known. As small intestinal cavernous lymphangiomas are benign lesions and have various clinical presentations, it is difficult to preoperatively diagnose and be associated with intussusception. In addition, patients had a good prognosis after partial resection of the small intestine in the literature. To the best of our knowledge, this is the first English report of intussusception caused by jejunal submucosal lymphangioma in an adult. Our purpose is to report a case and highlight this tumor within variable symptoms, the differential diagnosis for jejunal intussusception, and to identify computed tomography (CT) may help with preoperative diagnosis.

## Case report

A 32-year-old woman presented with abdominal pain and vomiting for 10 days, with a history of cesarean sections in 2015 and 2017, without family history of gastrointestinal tumors.

Vital signs remained stable and within normal ranges. Abdominal examination revealed tenderness and a 5 cm × 5 cm abdominal mass on the left side of the abdomen, without signs of peritoneal irritation.

**Figure 1:**
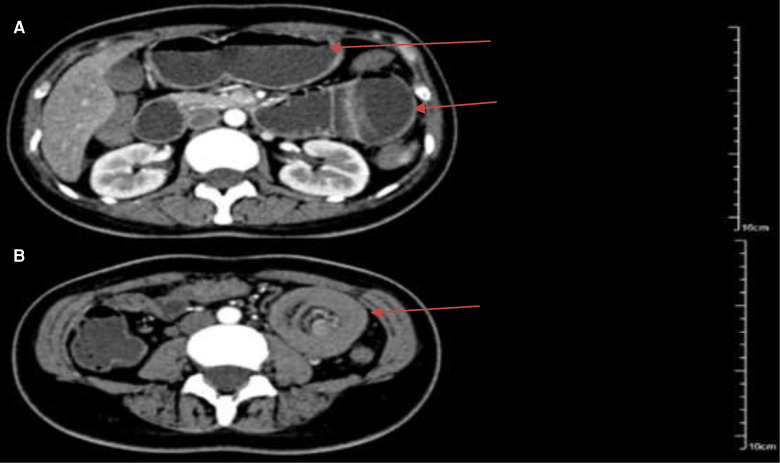
Arterial phase of abdominal contrast-enhanced computed tomography showed jejunal intussusception of the left-sided upper abdomen with concentric circle-like changes. (**A**) The intestinal canal of the upstream small intestine was dilated, and the outer intestinal wall was edematous and thickened (arrows); (**B**) there was the presence of the blood vessels and part of the mesenteric fat within a loop of jejunum which suggested intussusception with the jejunal lesion (arrow).

**Figure 2:**
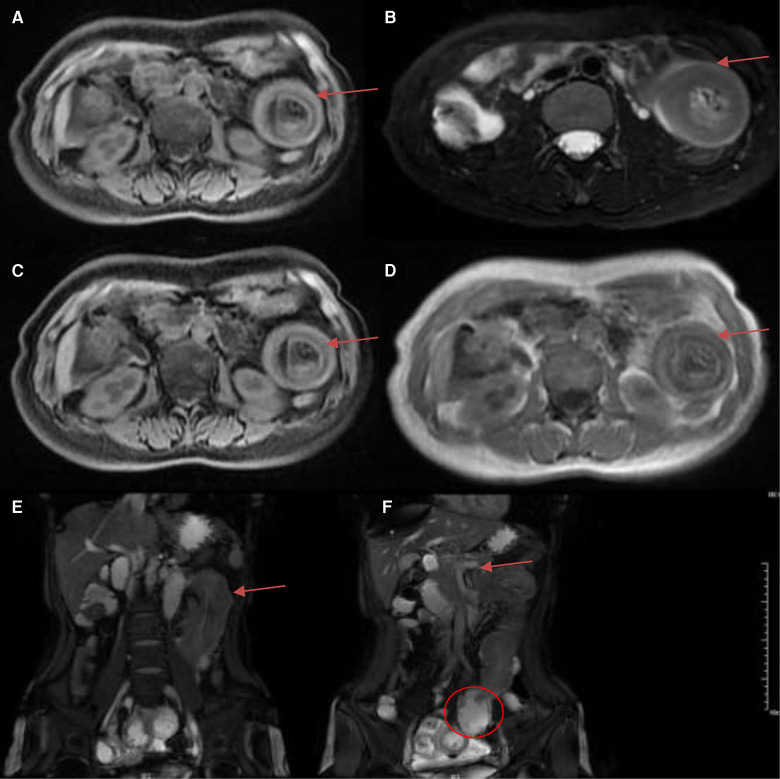
Magnetic resonance imaging (MRI) demonstrated jejunal intussusception of the left-sided upper abdomen with concentric circle-like changes. (**A**) T1 FS (arrow); (**B**) RTr OAx T2 fs (arrow); (**C**) WATER: Ax LAVA-Flex (arrow); (**D**) InPhase: Ax LAVA-Flex (arrow); (**E**) BH Cor 2D Fiesta Shim showed the intussusception of the long segment of jejunum from the pelvis to the left-sided upper abdomen, with concentric circle-like changes about 14.2 cm (arrows); (**F**) BH Cor 2D Fiesta Shim showed the thickening of the proximal jejunum intestinal wall with the thickest part about 2.6 cm, and the lumen was narrowed with peripheral exudation (arrow). The distal segment of the jejunum and ileum were dilated, with fluid accumulation and slightly thickened walls (circle).

**Figure 3:**
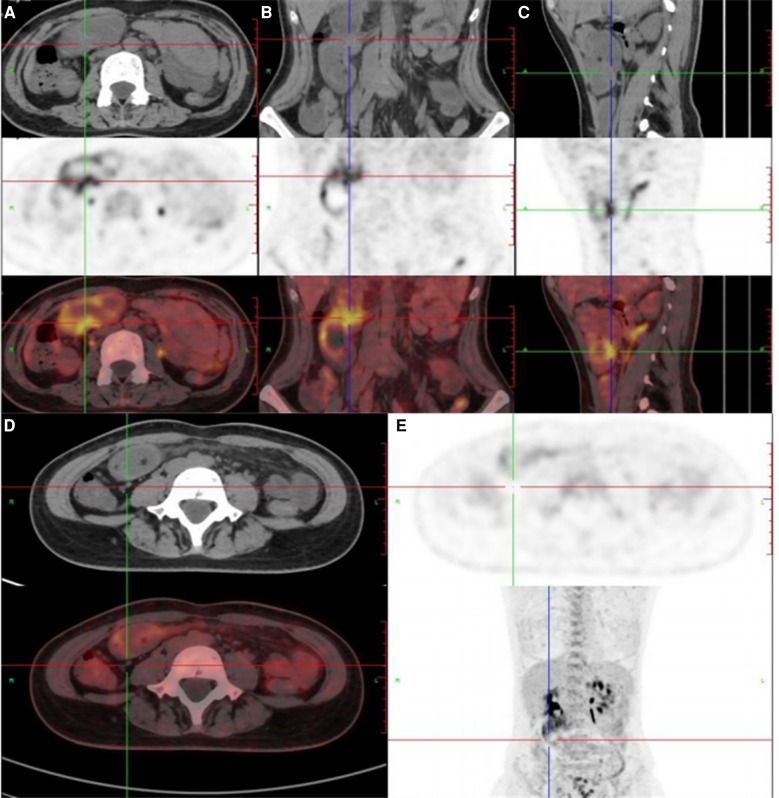
(**A–C**) 18F-FDG positron emission tomography/computed tomography showed that the distal intestinal metabolic activity increased unevenly; (**D,E**) multiple small lymph nodes were around the intestine without abnormal metabolic activity.

Laboratory tests showed that White blood cell was 6.54 × 10^9^/L, Neutrophil percentage was 87.4%, Hemoglobin was 10.3 g/dl, Amylase was 141 U/L, and *D* dimer was 630 ng/ml. Tumor biomarkers were not known. Contrast-enhanced CT of the abdomen showed possible small intestinal intussusception of the left-sided upper abdomen. There was the presence of fat within a loop of the jejunum which suggested intussusception with a jejunal lesion and a small amount of pelvic fluid (Figure 1). Magnetic Resonance Imaging (MRI) demonstrated thickening of the proximal jejunum intestinal wall (Figure 2). 18F-FDG positron emission tomography/computed tomography (18F-FDG PET/CT) showed that the distal intestinal metabolic activity increased unevenly, and multiple small lymph nodes were found around the intestine without abnormal metabolic activity (Figure 3).

**Figure 4:**
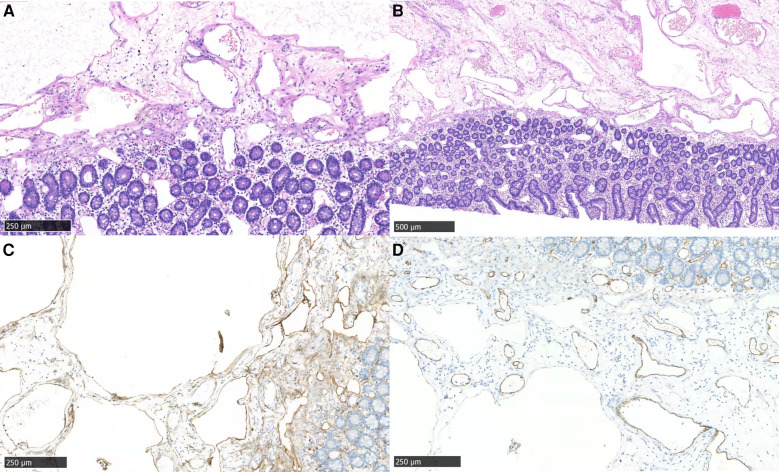
Microscopic features of the resected tumor. (**A**) morphology showed cysts in mucosal lamina propria and submucosa (hematoxylin-eosin staining, ×40); (**B**) mucosal lamina propria and submucosa of the jejunum (hematoxylin-eosin staining, ×20); (**C,D**) the endothelial cells were partially positive for D2-40 (**C**) and CD31 (**D**) (×40).

We would like to use laparoscopy, but we did not choose minimally invasive surgery because of the relatively limited abdominal space caused by intestinal obstruction. Jejuno-jejunal intussusception was identified and reduced, about 15 cm distal from the Treitz ligament. Segmental jejunal resection and ligation of mesenteric vessels from the distal 5 cm to the proximal 5 cm of the intussusception was performed, followed by side-to-side anastomosis. A soft, pedunculated polyp was found inside the wall of the jejunum. The stalk of the polyp and the entire length of the intestine was examined without any additional lesions found. The gross examination of this resected segment showed a 9 cm × 5 cm × 2 cm gray and red soft polyp confined to the submucosa. Pathology showed cavernous lymphangioma of the small intestine with secondary intussusception. Necrosis, hemorrhage, ulcer formation, and acute suppurative inflammation were in the intestinal wall. There was no tumor seen in lymph nodes (0/39). Microscopically, cysts were identified in mucosal lamina propria and submucosa layers (hematoxylin-eosin staining). The endothelial cells were partially positive for D2-40, CD31, CD34 (Figure 4), desmin, and elastin in immunohistochemical staining.

The patient was hospitalized for 11 days after surgery. At the 3-month follow-up, the abdominal CT looked the same as the one after partial bowel resection. At the 1-year follow-up, the patient was asymptomatic, her hemoglobin was normal and the gastrointestinal endoscopy showed smooth duodenal mucosa.

## Discussion

From 1940 to 1961, Viar reviewed 9 lymphangiomas in 52 cases of mesenteric cysts, mostly in the ileum and mesentery (8). Hanagiri reviewed 34 cases of small intestinal lymphangioma in the Japanese language literature from 1967 to 1990 (5). Gareth Morris-Stiff reviewed 19 case reports, 8 of which were Asian (9). Šileikis A et al. suggested that abdominal lymphangiomas occurred more frequently in young men (7). There were 4 jejuno-jejunal and 2 ileo-ileal intussusception cases secondary to small intestinal lymphangioma in adults but were not cavernous lymphangiomas (10–15). Wan YL et al. reported the first case of cystic lymphangioma of the cecal intussusception in an adult (16).

The authors searched Pubmed, Clinical Key, and Web of Science for English literature and CNKI, WAN FANG, and CQVIP for Chinese literature from inception up to January 1, 2022, for “lymphangioma” and “small intestine” as keywords. Patients under 18 years old were excluded, and those whose pathology showed “cavernous lymphangioma” were included. There were 23 cases in English literature from 1961 to 2021 (5, 8, 9, 17–33). Women account for 56.5% (13/23) of all cases, young people who were under 50 years old made up 56.5% (13/23) of them, there were 52.2% (12/23) cases whose lesions were located in the jejunum and there were 47.8% (11/23) Asian patients (Supplementary Table S1). There were 36 Chinese patients from 1951 to 2020 (Supplementary Table S2). 66.7% (24/36) of them were males, 77.8% (28/36) were under 50 years old, and 69.4% (25/36) were located in the jejunum. Only 3 of them had English abstracts, so most Chinese cases could not be reviewed in English.

The etiology of lymphangioma is unclear. Congenital lymphangiomas arise from abnormal embryonic development of the lymphatic system with gradual accumulation of lymph. In adults, lymphangiomas may develop secondary to lymphatic duct injury caused by infection, tumor, surgery, trauma, or radiation (34). We reported one case and collected three cases with a history of surgery (19, 27, 33), and two patients had tumor history (25, 32). Lymphangioma may be associated with PI3K/AKT/MTOR Signaling Pathway, Wnt/β-Catenin Signaling Pathways, and VEGF-C (35). Histopathologically, Wegner classified three types of cavernous lymphangiomas: simple, cavernous, and cystic lymphangiomas (36). Cavernous lymphangiomas have dilated and enlarged lymphatic lumens that are often connected to adjacent normal lymphatic spaces (23). Immunohistochemical markers help with diagnosis, D2-40 is a lymphatic endothelial cell marker, while CD31 and CD34 represent vascular endothelial cells.

Clinical presentation is nonspecific. It may be asymptomatic. Sometimes the mass grows slowly and aggressively, encasing and compressing the surrounding organs causing symptoms, and sometimes complications cause acute abdominal disease: torsion, intussusception, or rupture of cystic structures leading to acute peritonitis or intestinal obstruction. Invasion of the intestinal wall can cause gastrointestinal bleeding (8).

Preoperative diagnosis is difficult. However, endoscopy, ultrasound, CT, MRI, and PET/CT can help us. Patients with chronic black stools or abdominal masses can have lesions detected by endoscopy, and the development of small intestinal endoscopy is very helpful, while for patients with acute abdomen are difficult to use endoscopy. The septa in a cystic mass are usually more accurately delineated by ultrasound than by other techniques. But the accuracy of preoperative ultrasound localization and diagnosis is low (16). On CT scan, intussusception presents a typical intestinal-within-intestinal appearance with a pooled accuracy of 77.8% (37). And lymphangiomas present as unilocular or multilocular lesions with contrast enhancement of the cyst walls and septa, whose density is close to water (38). Cystic hypodense lesions with thin cyst wall, homogeneous density, long T1T2 signal, and compression lipid high signal are often present on MRI scans. PET/CT can detect hypermetabolic tumor cells to identify malignant tumors (25). According to our patient's PET/CT, we consider jejunal malignant lesions, infectious lesions, Kaposiform Hemangioendothelioma, and malignancies with mesenteric panniculitis as the differential diagnosis (39). So the resection margin should be from the distal 10 cm–15 cm to the proximal 10 cm–15 cm of the intestinal segment including the tumor, with ligation of mesenteric vessels and removal of the associated mesentery, regional lymph nodes, and adjacent organs the lesion involved. Nuclear imaging of lymphatic vessels and lymphangiography have a direct diagnostic role in defining the lesion, and detecting lymphatic patency and can be used to assess the function of the lymphatic system (40).

Most patients undergo surgical resection of the lesion and part of the small intestine, and pancreaticoduodenectomy is performed for lesions involving the head of the pancreas (28, 29). In this case, when the patient came to the emergency room, she was not allowed to eat or drink and was instead given intravenous fluids for hydration. After using omeprazole sodium for injection, lactulose, and glycerin enema, her symptoms disappeared and started to pass gas. Her family chose conservative treatments then they went to the department of gastroenterology for MR and PET/CT. Until she ate something and then presented with abdominal pain and vomiting again, they agreed to surgery. With the popularity of endoscopy, successful microscopic resection of the lesion has also been performed instead of surgery (17, 33). Surgical resection of part of the small intestinal after endoscopic labeling was successful in some cases (9, 26). Atsushi Kohga et al. used single-incision laparoscopic-assisted surgery (SILS) for a small intestinal intussusception caused by a cystic lymphangioma (14). The prognosis is good, without a case of recurrence reported in the literature.

## Conclusion

In summary, we report a rare adult case with jejuno-jejunal intussusception caused by a small intestine cavernous lymphangioma. Most of the cases are under 50 years old, men, Asian, and located in jejunum. Preoperative diagnosis is difficult, but CT, ultrasound, and PET/CT are helpful. Surgical resection is a good curative method without a case of recurrence reported in the literature. The final diagnosis of cavernous lymphangioma will be made by histological examination.

## Patient perspective

The patient was uninterested in follow-up or learning about the condition. She had the same abdominal pain after exercising when she was 14 years old. But there was no abnormality on gastroscopy and the pain disappeared soon. However, the pain existed for 10 days without eating anything. She was particularly impressed that she had many CT scans, but the diagnosis was not verified. She hoped the doctor would confirm the diagnosis as soon as possible so she could feel easy.

## Data Availability

The original contributions presented in the study are included in the article/**Supplementary Material**, further inquiries can be directed to the corresponding author/s.
