# Supplementary material for: Case report: Submucosal cavernous lymphangioma causing jejuno-jejunal intussusception in an adult
Source: Front Surg. 2022 Sep 22;9:953840. doi: 10.3389/fsurg.2022.953840 (PMC9632977; doi:10.3389/fsurg.2022.953840)
Supplement: Supplementary file 1 [file Datasheet1.docx]

Supplementary Material

# Supplementary Data

# Supplementary Figures and Tables

## Supplementary Figures

**
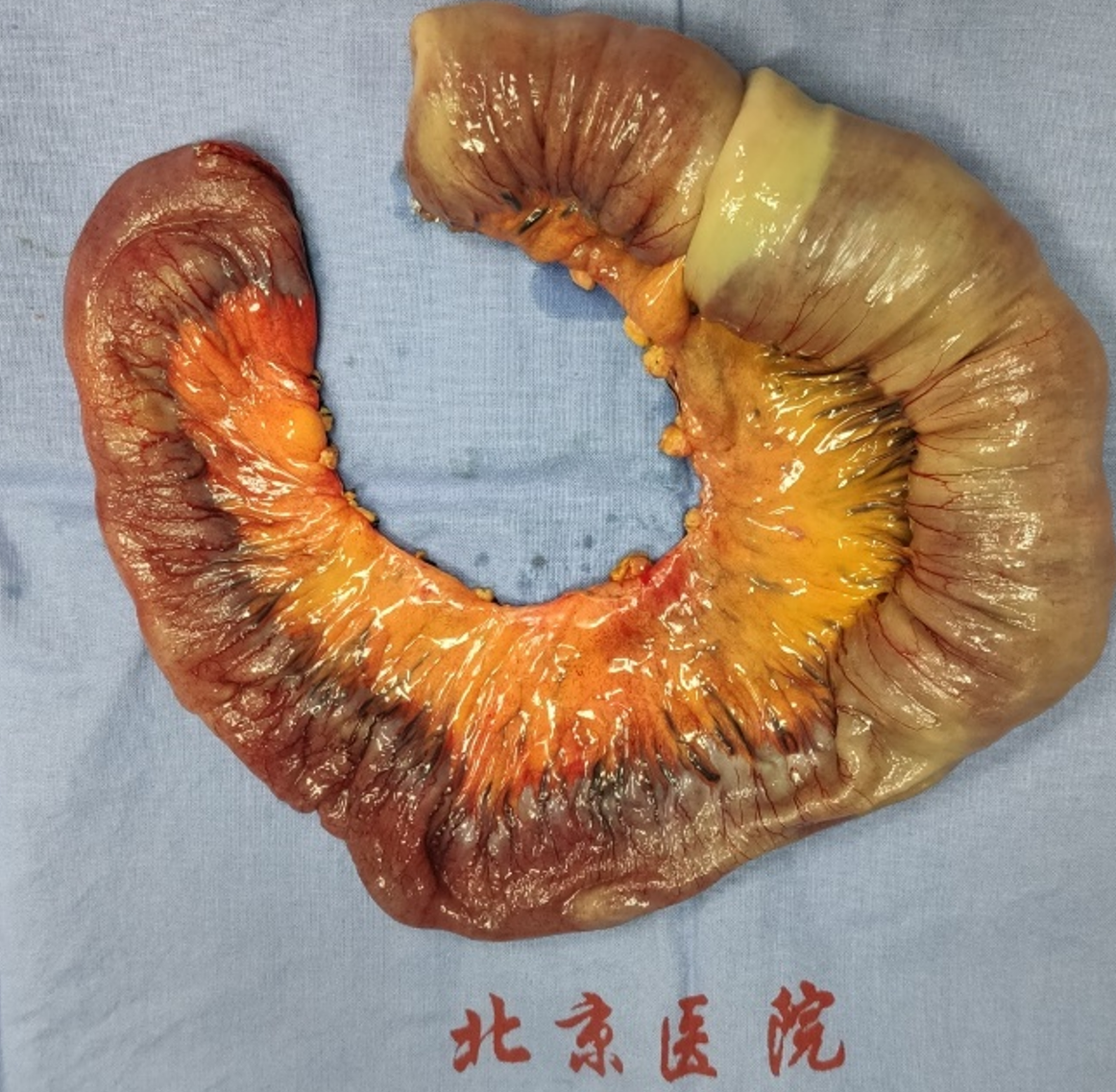
**

**Supplementary Figure 1.** Gross picture of jejuno-jejunal intussusception, 15 cm distal from the Treitz ligament, was identified and reduced.

## Supplementary Tables

| Author | Year | Gender | Age | | country | Presentation | Hb(g/dL) | Location | Mesentery |
| --- | --- | --- | --- | --- | --- | --- | --- | --- | --- |
| João (1) | 2021 | F | | 29 | Portugal | M | 5.5 | J | - |
| Tan (2) | 2020 | F | | 29 | China | M 1y | 6.8 | J | - |
| Rojas (3) | 2018 | F | | 71 | USA | P 1m, mass | NS | SI | + |
| Hong (4) | 2015 | F | | 53 | Korean | Asymptomatic | 13.5 | SI | + |
| Xu(5) | 2014 | M | | 63 | China | M 1year | 6.7 | J | - |
| Vennarecci (6) | 2013 | M | | 42 | Italy | P, mass | normal | SI | + |
| Tang (7) | 2013 | F | | 38 | USA | M, A | NS | J | - |
| MONÇÃO (8) | 2013 | M | | 68 | Brazil | M, A | NS | D and J | + |
| Morris-Stiff (9) | 2011 | F | | 34 | USA | A, dyspnoea | 6 | J | + |
| Hwang (10) | 2009 | M | | 71 | Korea | M | NS | J | + |
| Huang (11) | 2008 | M | | 57 | USA | M | 7.6 | J | - |
| Christofi (12) | 2007 | F | | 24 | UK | P | NS | SI | + |
| Fujishiro (13) | 2002 | M | | 40 | Japan | Mass, fever | NS | D, PH, RS | - |
| Chung (14) | 2000 | M | | 22 | Korea | P | NS | SI | + |
|  | 2000 | M | | 19 | Korea | P | NS | J | + |
|  | 2000 | F | | 28 | Korea | NS | NS | SI | + |
| Seki (15) | 1998 | F | | 54 | Japan | P, Fever, mass | NS | J | + |
| Barquist (16) | 1997 | F | | 33 | Panama | A, Dyspnoea | 5.5 | J | - |
| Ikura (17) | 1994 | M | | 76 | Japan | M | 4.5 | D | - |
| Hanagiri (18) | 1992 | M | | 53 | Japan | P, nausea | 11 | J | - |
| Davis (19) | 1987 | F | | 53 | USA | P | NS | D | - |
| Viar (20) | 1961 | F | | 22 | USA | P, fever 36h | 11.8 | I | + |
|  | 1961 | F | | 35 | USA | P, fever | 12 | I | + |

**Supplementary Table 1.** 23 English cases of small intestinal cavernous lymphangioma. Abbreviations: A, anemia; d, day; D, duodenum; E, endoscopy; FOBT, fecal occult blood test; Hb, hemoglobin g/dL; HDL, hepatoduodenal ligament I, ileum; J, jejunum; m, month; M, melena; NS, not stated; P, abdominal pain; R, radiography; RBC, red blood cell; SI, small intestine; V, vomiting; w, week; y, year; PH, pancreatic head; RS, retroperitoneal space.

| Author | Year | Gender | Age | Presentation/time | Hb(g/dL) | Location | Mesentery |
| --- | --- | --- | --- | --- | --- | --- | --- |
| Zheng W | 2020 | M | 47 | M | N | J | - |
| Chai H | 2020 | F | 54 | P, V | 10.9 | J | + |
| Yang J | 2019 | M | 26 | M | 3.6 | I | - |
| Yang W | 2018 | F | 44 | M | 8.3 | J | - |
| Wang J | 2018 | F | 31 | A | 3.8 | J | - |
| Dong X | 2018 | M | 54 | mass | N | I | + |
| Zhao X | 2016 | F | 76 | M | 3.7 | J | - |
| Huang Q | 2015 | F | 36 | M | 5.6 | J | - |
| Zhang W | 2010 | M | 39 | M | 5.6 | J | - |
| Yang W | 2009 | M | 35 | P | NS | J | + |
| Shen X | 2008 | F | 39 | M | NS | D, HDL | - |
| Zheng C | 2007 | M | 32 | P 5d | NS | SI | + |
| Zhong H | 2005 | F | 25 | P | NS | I | - |
| Sun Y | 2005 | M | 22 | P 2m | 13.0 | I | + |
| Meng Y | 2005 | M | 27 | P 2d | 13.7 | J | - |
| Jiang L | 2005 | M | 39 | mass 1w | NS | J | - |
|  |  | M | 30 | mass 2w | NS | SI | + |
|  |  | M | 48 | P 1y | NS | J | - |
|  |  | M | 58 | mass 1w | NS | J | - |
| Cheng B | 2003 | M | 22 | M 5y | 3.5 | J and I | + |
| Jiao F | 2002 | F | 58 | mass 6m | NS | J | + |
| Shao L | 1999 | M | 59 | A, mass 1y | NS | J | + |
| Zhang S | 1997 | M | 26 | P 1w | 9.5 | I | + |
| Chen Y | 1997 | M | 46 | P 2m | NS | J | + |
| Cui L | 1996 | M | 21 | P 5d | NS | I | - |
| Su G | 1994 | M | 39 | P 6d | 16.2 | J | - |
| Qi L | 1993 | M | 37 | M 18y | 5.6 | J | - |
| Li B | 1992 | M | 21 | P, mass 4y | 11.5 | J | - |
| Zhang S | 1991 | M | 24 | M 2m | 5.7 | J | + |
| Ren H | 1988 | M | 33 | mass 2m | NS | J | + |
|  |  | F | 18 | P 10y | NS | J | + |
| Zhao J | 1987 | F | 55 | mass 4y | NS | I | + |
| Xin W | 1987 | M | 67 | P 8d | 14.5 | J | + |
| Qian Z | 1982 | M | 21 | P 3d | NS | I | + |
| Ye T | 1981 | F | 48 | M 1w | 3.6 | J | - |
| Wang Y | 1951 | F | 28 | P, mass 1d | NS | J | + |

**Supplementary Table 2.** 36 Chinese cases of small intestinal cavernous lymphangioma. Abbreviations: A, anemia; d, day; D, duodenum; E, endoscopy; FOBT, fecal occult blood test; Hb, hemoglobin g/dL; HDL, hepatoduodenal ligament I, ileum; J, jejunum; m, month; M, melena; NS, not stated; P, abdominal pain; R, radiography; RBC, red blood cell; SI, small intestine; V, vomiting; w, week; y, year.

**References**

1. Joao M, Gravito-Soares E, Lopes S, Amaro P. Jejunal cavernous lymphangioma: successful endoscopic treatment of a rare cause of small bowel bleeding. Ann Gastroenterol. 2021;34(6):891.

2. Tan B, Zhang SY, Wang YN, Li Y, Shi XH, Qian JM. Jejunal cavernous lymphangioma manifested as gastrointestinal bleeding with hypogammaglobulinemia in adult: A case report and literature review. World J Clin Cases. 2020;8(1):140-8.

3. Rojas CL, Molina GA. Lymphangioma cavernous of the small bowel mesentery, an infrequent cause of acute abdomen in adult. J Surg Case Rep. 2018;2018(2):rjy018.

4. Hong IT, Cha JM, Lee JI, Joo KR, Baek IH, Shin HP, et al. [A Case of Cavernous Lymphangioma of the Small Bowel Mesentery]. Korean J Gastroenterol. 2015;66(3):172-5.

5. Xu X, Liu W, Zheng C. A rare cause of repeated gastrointestinal bleeding. Mesenteric cavernous lymphangioma. Gastroenterology. 2014;146(4):e11-3.

6. Vennarecci G, Ceribelli C, Laurenzi A, Moroni E, Ettorre GM. Giant cavernous mesenteric lymphangioma in adult. Updates Surg. 2013;65(4):317-9.

7. Shou-jiang Tang FB. Small Bowel Lymphangioma. Video Journal and Encyclopedia of GI Endoscopy. 2014;1(3):663-5.

8. Moncao CR. Approach to upper digestive hemorrhage with diagnosis of cavernous lymphangioma. Arq Bras Cir Dig. 2013;26(1):69-70.

9. Morris-Stiff G, Falk GA, El-Hayek K, Vargo J, Bronner M, Vogt DP. Jejunal cavernous lymphangioma. BMJ Case Rep. 2011;2011.

10. Hwang SS, Choi HJ, Park SY. Cavernous mesenteric lymphangiomatosis mimicking metastasis in a patient with rectal cancer: a case report. World J Gastroenterol. 2009;15(31):3947-9.

11. Huang Q, Minor MA, Weber HC. Clinical challenges and images in GI. Diagnosis: Cavernous lymphangioma of the jejunum. Gastroenterology. 2009;136(4):1170, 465.

12. Christofi N, Hextall A. Cystic cavernous lymphangioma of the mesentery in a patient with Cowden syndrome. J Obstet Gynaecol. 2007;27(3):329-30.

13. Fujishiro M, Kamoshida T, Hotra S, Hirai S, Oka Y, Sato M, et al. Retroperitoneal lymphangioma with a duodenal lesion in an adult. J Gastroenterol. 2002;37(5):381-6.

14. Chung JH, Suh YL, Park IA, Jang JJ, Chi JG, Kim YI, et al. A pathologic study of abdominal lymphangiomas. J Korean Med Sci. 1999;14(3):257-62.

15. Seki H, Ueda T, Kasuya T, Kotanagi H, Tamura T. Lymphangioma of the jejunum and mesentery presenting with acute abdomen in an adult. J Gastroenterol. 1998;33(1):107-11.

16. Barquist ES, Apple SK, Jensen DM, Ashley SW. Jejunal lymphangioma. An unusual cause of chronic gastrointestinal bleeding. Dig Dis Sci. 1997;42(6):1179-83.

17. Ikura Y, Hashimoto T, Takamine Y, Tani T, Konishi Y, Uchida H, et al. Lymphangioma of the duodenum: report of a case. Surg Today. 1994;24(2):160-3.

18. Hanagiri T, Baba M, Shimabukuro T, Hashimoto M, Takemoto H, Inoue A, et al. Lymphangioma in the small intestine: report of a case and review of the Japanese literature. Surg Today. 1992;22(4):363-7.

19. Davis M, Fenoglio-Preiser C, Haque AK. Cavernous lymphangioma of the duodenum: case report and review of the literature. Gastrointest Radiol. 1987;12(1):10-2.

20. Viar WN, Scott WF, Jr., Donald JM. Mesenteric cavernous lymphangiomata: brief review and report of two cases. Ann Surg. 1961;153:157-60.
